# Membrane flexibility, free fatty acids, and the onset of vascular and neurological lesions in type 2 diabetes

**DOI:** 10.1186/s40200-016-0235-9

**Published:** 2016-04-27

**Authors:** Rob N. M. Weijers

**Affiliations:** Teaching Hospital, Onze Lieve Vrouwe Gasthuis, Amsterdam, The Netherlands

**Keywords:** Membrane flexibility, Type 2 diabetes, Free fatty acids, Diabetic nephropathy, Diabetic retinopathy

## Abstract

Free fatty acids released from human adipose tissue contain a limited amount of non-esterified poly-*cis*-unsaturated fatty acids. In cases of elevated plasma free fatty acids, this condition ultimately leads to a shift from unsaturated to saturated fatty-acyl chains in membrane phospholipids. Because this shift promotes the physical attractive van der Waals interactions between phospholipid acyl chains, it increases stiffness of both erythrocyte and endothelial membranes, which causes a reduction in both insulin-independent and insulin-dependent Class 1 glucose transporters, a reduction in cell membrane functionality, and a decreased microcirculatory blood flow which results in tissue hypoxia. Against the background of these processes, we review recently published experimental phospholipid data obtained from *Drosophila melanogaster* and from human erythrocytes of controls and patients with type 2 diabetes, with and without retinopathy, along the way free fatty acids interfere with eye and kidney function in patients with type 2 diabetes and give rise to endoplasmic reticulum stress, reduced insulin sensitivity, and ischemia.

## Background

To understand, in more detail, the basic concepts of type 2 diabetes pathophysiological mechanisms, we summarize: the structure of biological membranes, the importance of membrane flexibility, the effect of increased plasma free fatty acids (FFAs) on membrane flexibility, the relationship between increased FFAs and both the development of type 2 diabetes and microvascular complications. In conclusion, we suggest that a long period of increased FFAs must be avoided to prevent or postpone diabetes-related microvascular complications.

### Biological membranes

Compartmentalization, a cell-like structure that separates the cytoplasm of a cell from the surroundings, is one of the six basic properties shared by all living cells [1]. After 4 billion years of evolution, these cell-like structures of the earliest protocells evolved to ‘modern’ cells enclosed by membranes consisting of phospholipids, the major constituents of the biological membranes of which the glycerol-based phospholipids are the major class of naturally occurring phospholipids. In most cases, glycerophospholipids have a saturated fatty acid at position 1 and an unsaturated fatty acid at position 2 of the glycerol [2]. Saturated fatty acids possess essentially linear alkyl chains with no double bonds. Conversely, double bonds in unsaturated fatty acids are nearly in the *cis* configuration, which produces a bend in the fatty-acid chain. Molecules like palmitoleic acid (C16:1) and oleic acid (C18:1) are bent at the *cis* double bond, and the two chain parts form an angle of 133° [3–6]. This bend has important consequences for the structure and functionality of biological membranes. Saturated fatty acid chains can pack closely together, whereas unsaturated fatty acids prevent such close packing and produce flexible, fluid aggregates. The two fatty acid chains of a phospholipid molecule (the hydrocarbon chain region) yield a roughly cylindrical shape. The hydrocarbon chain region is characterized by an area (A), that is the surface of the cross-section of the cylindrical part. Note that unsaturation compared to saturation results in a larger cross-section value for A [7, 8].

Replacement of saturated acyl chains into mono- or poly-*cis*-unsaturated acyl chains of a model phospholipid membrane increases the area (A) by approximately 15 %, which involves an increase in the interchain distance of the fatty acyl chains. Because the attractive van der Waals forces diminish as 1/r^6^, the increase in the interchain distance decreases these physical attractive intermolecular forces between the fatty acyl chains leading to an increased membrane flexibility and a decreased membrane rigidity [9, 10]. The unsaturation index (UI) is a useful parameter for describing the flexibility of a biological membrane and is calculated by multiplying the mean number of *cis* double bonds per fatty-acid residue by 100 [11]. Arachidonic acid and docosahexaenoic acid are key fatty acids; a minimal increase in the percentage of arachidonic acid in phospholipids tails improves membrane flexibility due to its four double bonds. A similar effect is seen for docosahexaenoic acid with its six unsaturated bonds. In other words, an increase in *saturated* fatty acids of membrane phospholipids results in a decrease of membrane flexibility, and is marked by a reduction of UI.

It is noteworthy that the reversibility of this process depends on the physical background of the Lennard-Jones potential, a mathematically straight forward model that describes the interaction between a pair of neutral atoms or molecules. Importantly, calculations using a statistical thermodynamic methodology and Langevin dynamics indicated that the results of these computer simulations are in qualitative agreement with the results of experimental studies [12, 13].

### The importance of membrane flexibility

There are at least two senses why membrane flexibility is important. First, it affects the insertion of glucose transporters (GLUTs) into cell membranes. GLUT1 is a monomeric protein with 12 transmembrane helical segments [14]. During the insertion machinery, the transporter protein traverses the plasma membrane 12 times in a zigzag fashion, before initiating the folding necessary to form the three-dimensional structure. One molecule GLUT1 with a mean cross-section area of about 1100 Ǻ^2^ covers an area of about 17 molecules of a phosphatidylcholine bilayer with saturated fatty acyl chains [15], which requires a high membrane flexibility for pore formation. GLUT4 is inserted into the membrane of intracellular vesicles which demands flexibility of the vesicular membrane. The GLUT4 containing vesicles take part in a fusion process with the cell membrane. In the final stage of this process, fusion proteins induce bending of the plasma membrane bilayer and drive fusion pore formation, which proceeds more smoothly at an increased flexibility of both membranes [16, 17]. Thus, reduced membrane flexibility causes a reduction in all Class 1 glucose transporters which, in turn, reduces the glucose flux, and increases the plasma glucose concentration. Thus, high membrane flexibility represents a key determinant in glucose transport due to its influence on all Class 1 GLUT proteins.

Second, membrane stiffness induces tissue hypoxia. The erythrocyte membrane is compositionally very similar to the vascular endothelium [18]. In support of this, the UI of red cell membrane phospholipids reported for healthy controls was 155.4 [19], while the reported UI for cultured endothelial cells from human umbilical cord veins was 148.2 ± 6.3 [20]. Therefore, it is likely that if the erythrocyte membrane is selectively affected in type 2 diabetes, the endothelium could also be affected with the expected consequences of vascular disfunction [21, 22]. In capillaries in which the size of red blood cells is of the same order of magnitude as the lumen (about 8 μm) flexibility is an important determinant of blood flow. Increased stiffness of both membranes may decrease the microcirculatory blood flow. This reduction of the microcirculatory blood flow causes reduced oxygen uptake in tissues and thus hypoxia [23, 24]. Eukaryotic cells use oxygen as the final electron acceptor of the oxidative phosphorylation step, to drive ATP synthesis. And that means decreases in O_2_ may be an important player in the impaired ATP production of (skeletal muscle) mitochondria in type 2 diabetes [25–28]. In more general terms, a reduction in membrane flexibility affects the continuous flux of ATP production (*i.e*., energy) required for a proper functioning of all living cells. For the reader’s mind-set, a single cell consumes around 10 *million* molecules of ATP every second, giving a total turnover of ATP of around 80 kg per day [1].

### Increase of free fatty acids affects membrane flexibility

Type 2 diabetes and its prediabetic phase, gestational diabetes mellitus, and obesity are characterized by increased plasma FFAs [29–35]. As discussed in the aforementioned paragraph, the cause of this phenomenon is a reduction of GLUTs which reduces the ATP production *via* the coupled metabolic reaction chain comprising glycolysis, tricarboxylic acid cycle, and oxidative phosphorylation. This alarming situation demands for an increase in ATP *via* ß-oxidation of circulating FFAs and stimulates so adipose-tissue lipolysis. We propose the latter is the classic trigger for the onset of type 2 diabetes and its vascular lesions. It is currently widely believed that the release of fatty acids in humans is proportional to the fatty-acid content in adipose tissue [35]. The released FFA-pool from human white cells had approximately 110- and 9-fold decreased percentages of docosahexaenoic acid (C22:6) and arachidonic acid (C20:4), respectively, compared with the human serum pool (Table 1). We calculated the UIs of released FFAs from human white fat cells [36] and serum FFAs in healthy controls [37] and, as was to be expected, the former was substantially lower (85.5 and 191.9, respectively; Δ = 55.4 %) (Table 1). Thus, an increased release of FFAs from adipose tissue into the circulation elevates the plasma concentration of *saturated* fatty acids. So, a shift is forced from unsaturated to saturated fatty-acyl chains in phospholipids of both the erythrocyte membrane and the vascular endothelium [10]. Summarized, we have now a conceptual argument that increased FFAs cause a reduction in membrane flexibility which, in turn, affects oxygen transport and, thus, a continuous flux of ATP production (*i.e*., energy) necessary for a proper functioning of living cells. A number of papers have reported examples of reduced membrane flexibility; a review by Cho et al. indicated that patients with type 2 diabetes exhibited reduced erythrocyte deformability compared to healthy controls [38].

**Table 1:** Unsaturation index of non-esterified fatty acids released from human white fat cells, and human serum lipids of healthy subjects

Fatty acids	Released from white fatt cells^a^		Serum lipids^b^	
	mol% of total fatty acids	total number of double bonds	mol% of total fatty acids	total number of double bonds
C12:0	0.61	-		
C14:0	3.51	-		
C15:0	0.40	-		
C16:0	24.97	-		
C18:0	6.14	-		
C20:0	0.08	-		
C22:0	0.02	-		
∑ SFA			27.05	-
C14:1	0.26	0.26		
C16:1	4.40	4.40		
C17:1	0.36	0.36		
C18:1	40.70	40.70		
C20:1	0.58	0.58		
C22:1	0.03	0.03		
∑MUFA			23.60	23.60
C18:2 n-6	15.74	31.49	27.47	54.94
C20:2 n-9	0.02	0.03		
C20:2 n-6	0.23	0.46		
C18:3 n-3	0.73	2.20	2.48	7.44
C18:3 n-6	0.05	0.15		
C20:3 n-6	0.21	0.64		
C20:3 n-3	0.03	0.08		
C20:4 n-6	0.51	2.04	4.48	17.92
C20:4 n-3	0.04	0.14		
C22:4 n-6	0.08	0.33		
C20:5 n-3	0.09	0.45	1.56	7.80
C22:5 n-6	0.01	0.04		
C22:5 n-3	0.09	0.45		
C22:6 n-3	0.12	0.70	13.36	80.16
Total	100.00		100.00	
UI		85.53		191.86

For conversion to mol%, ^a^the data listed by Raclot et al. [36] were multiplied by: (MW_FFA_)^-1^ · 10^3^ · 0. 272, and ^b^those by Wang et al. [37] by 0. 142. The unsaturation index was calculated as the mean number of *cis* double bonds per fatty-acid residue multiplied by 100. *SFA* saturated fatty acid, *MUFA* monounsaturated fatty acid, *MW*, molecular weight

This concept is in accordance with the calculated UIs based on the data coming from a study on erythrocyte phospholipid and poly-*cis*-unsaturated fatty-acid (PUFA) composition in diabetic retinopathy [39]. Categorizing fatty acids in saturated fatty acids, mono-unsaturated fatty acids, and PUFAs, the phospholipid fatty-acid groups’ data from controls and patients with type 2 diabetes without and with retinopathy revealed a substantial time-dependent shift from unsaturation into saturation (Table 2). Accordingly, the calculated UI was substantially lower in individuals with type 2 diabetes without retinopathy than in healthy controls (134.3 and 155.4, respectively; Δ = 13.5 %), and the calculated mean UI for subjects with type 2 diabetes with mild, moderate, and severe diabetic retinopathy was substantially lower than in individuals with type 2 diabetes without diabetic retinopathy (123.4 and 134.3, respectively; Δ = 8.1 %) [19, 39]. The observed declining trend of UIs underlines the hypothesis that the onset of type 2 diabetes is characterized by a substantial increase of plasma *saturated* fatty acids which includes a decrease in UI and a concomitant decrease in membrane flexibility. The data are also in agreement with the notion that, after frank type 2 diabetes, an ongoing decrease of UI effectuates the development of vascular and neurological lesions, due to an ongoing decrease of membrane flexibility and functionality. It is interesting to note that (1) individuals with an impaired glucose tolerance test have a significantly increased FFA concentration compared to healthy controls [40], (2) the increase in FFAs is augmented after the onset of type 2 diabetes [40], and (3) patients with type 2 diabetes exhibited significantly reduced erythrocyte flexibility compared to healthy controls [41]. Thus, an increase in FFAs causes an increase in plasma *saturated* fatty acids which, in turn, creates an shift from unsaturation into saturation of biological membranes which affects their flexibility and functionality.

**Table 2:** Erythrocyte fatty acid composition of controls, individuals with type 2 diabetes without diabetic retinopathy, and individuals with type 2 diabetes with diabetic retinopathy^a^

Fatty acid category	Controls	Individuals with type 2 diabetes without retinopathy	Individuals with type 2 diabetes with retinopathy
SFAs (%)	41.83	43.99	46.61
MUFAs (%)	18.85	21.68	21.26
PUFAs (%)	37.97	31.92	29.47

*SFA* saturated fatty acid, *MUFA* monounsaturated fatty acid, *PUFA* poly-*cis*-unsaturated fatty acid

^a^Original data listed by Koehrer et al. [39]

This concept is inherent in all biological membranes. In this context, I would like to recall a recent report from the Academy of Medical Royal Colleges [42], which quite rightly states that complications of type 2 diabetes are common and affect all systems: diabetic retinopathy, nephropathy, neuropathy, microvascular complications, ulcers and sexual dysfunction.

A recent study demonstrated that *Drosophila melanogaster*’s phototransduction (the response to light) depends on the degree of saturation of its phospholipid membrane [43]. The proportion of polyunsaturated phospholipids in flies reared on a yeast diet, lacking PUFAs, was significantly reduced, but was rescued by adding a single type of PUFA (α-linolenic [18:3] or linoleic acid [18:2]) to the diet. Photoreceptors from yeast-reared flies showed a substantial increase in latency and time to peak light response compared to control flies. This phenomenon disappeared by adding either α-linolenic (C18:3) or linoleic acid (C18:2) to the diet. The authors underlined the reverse relationship between the mean number of *cis* double bonds per fatty-acid residue of a biological membrane and its stiffness [44, 45]. Another recent study [46] showed that deficiency of PUFAs strongly affected the synaptic transmission in the visual system of *Drosophila melanogaster* fly’s, which defects were rescued by diets containing ω-3 or ω-6 PUFAs alone or in combination. In contrast to Nyengaard et al., we propose that the capillary closure, observed by proliferative retinopathy in retinas from rats, results from membrane stiffness and hypoxia [24]. Data of these studies need to be carefully examined as the animal model results do not compulsorily translate into human medicine. However, mammalian retinal ganglion cells phototransduction cascades show similarity with fly visual transduction. It is noteworthy that the *Drosophila melanogaster* subgroup diverged from the cluster of the three major groups in the *melanogaster* group during the Late to Middle Eocene (35–45 million years ago) [47].

### Increased saturated fatty acids and development of type 2 diabetes

**Fig. 1:**
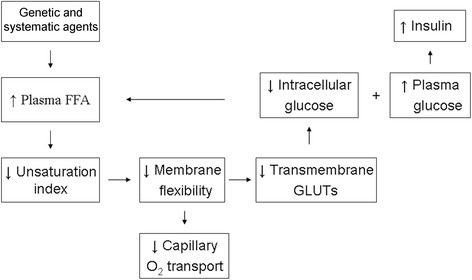
Proposed steps in the development of type 2 diabetes model. Although the results of genome-wide screen for type 2 diabetes susceptibility genes is still being debated, we hypothesize that the involved genes of the disease lead to an increase in plasma free fatty acids; FFA: free fatty acid; GLUT: glucose transporter

In a previous paper [10], we explained the consequence of increased fatty-acid exposure in relation to the risk of progression to type 2 diabetes (Fig. 1). Shortly, we hypothesized that the involved genes cause an elevation of plasma FFAs which leads to an increase in plasma saturated FFAs and, consequently, to a decrease in membrane phospholipid UI, as seen in erythrocytes. The result is an increase in membrane stiffness and a partial loss off all GLUTs. The consequence of this reasoning is a decrease in both glucose effectiveness and insulin sensitivity as seen in individuals with type 2 diabetes compared to healthy controls (Table 3). This hypothesis had to be demonstrated [48–51]. This process causes an increase in plasma glucose and insulin concentrations until ß-cell failure occurs and the condition of glucose tolerance gives way to frank type 2 diabetes.

**Table 3:** Values of glucose effectiveness (S_G_) and insulin sensitivity (S_I_) for minimal model

Units S_G_	Control subjects	Type 2 diabetes	*P*	Compart-ment	Tracher	Ref.
min^-1^	0.016 ± 0.001	0.010 ± 0.001	<0.01	one	no	48
	0.023 ± 0.012	0.013 ± 0.001	<0.05	one	no	49
	0.023 ± 0.012	0.016 ± 0.009	<0.001	one	no	50^a^
h^-1^	1.2 ± 0.16	0.81 ± 0.11	<0.001	one	no	51
	0.41 ± 0.04	0.33 ± 0.02	<0.001	two	^13^C	51
	0.52 ± 0.05	0.37 ± 0.02	<0.001	two	^2^H	51
Units S_I_						
10^-4^∙min^-1^∙(mU/L)^-1^	11.8 ± 2.6	6.7 ± 0.8	<0.01	one	no	49
	13.45 ± 11.12	5.31 ± 3.98	<0.01	one	no	50 ^a^
pmol∙L^-1^∙h^-1^	0.0062 ± 0.0006	0.0019 ± 0.0006	<0.01	one	no	51
	0.0082 ± 0.0012	0.0036 ± 0.0006	<0.001	two	^13^C	51
	0.0098 ± 0.0013	0.0042 ± 0.0008	<0.001	two	^2^H	51

^a^More than 10 years before the development of type 2 diabetes

The fundamental coincidence between the experimental *Drosophila melanogaster* data [43, 46] and the UI data from subjects with type 2 diabetes with and without retinopathy [19, 39] support our working hypothesis that saturated FFAs influence membrane flexibility and, consequently, membrane functionality.

Additionally, we discussed [52] the consequences of Liposin II infusions, as reported by Shuman’s group [53]. We calculated the UIs of Liposin II and serum FFAs in healthy controls and the former was substantially lower (162 and 191.9, respectively; Δ = 15.2 %), which is the principal cause of the decrease in all functional Class I glucose transporters. In other words, this decrease in glucose transporters became visible as the transmembrane “inhibition of glucose transport” [53].

We already mentioned the very similar composition of the vascular endothelium and the erythrocyte membrane. Consistent with this concept is the observation that an elevation of the circulating FFA concentration in lean healthy humans causes endothelial dysfunction, which is similar to that observed in obese individuals with reduced insulin sensitivity, and with or without type 2 diabetes [54]. Also, the authors suggested that it is this dysfunction, which over time is likely to increase greatly the risk of macrovascular disease. We can scarcely avoid the inference that a decrease in membrane flexibility, due to an increase in FFAs, is the underlying cause of the well-known relationship between endothelium dysfunction and type 2 diabetes [55].

### Microvascular complications

In the section ‘The importance of membrane flexibility’, we discussed an increase in plasma unsaturated fatty acids and its implications for diabetic retinopathy; however, increased plasma FFAs also play a crucial role in the relentless progress of diabetic kidney disease and affect function and survival of podocytes [56]. Previous studies of podocyte function showed results, which are in support of the working hypothesis; for example, derangements of the architecture of podocytes and glomerular endothelial cells have significant effects on kidney function in patients with type 2 diabetes [57]. We suggest that an increased stiffness of both podocyte cell membrane and glomerular endothelium the driving force is behind podocyte detachment and reduced endothelial cell fenestration in patients with type 2 diabetes.

Reperfusion of ischemic tissues is often associated with microvascular injury. These “activated” endothelial cells produce more reactive oxygen species. In 2009, a study examined the role of a large number of recently identified factors that contribute to podocytopathies in diabetic kidney disease and found a number are reactive oxygen species [58].

Decreased microcirculatory flow causes reduced oxygen uptake and endoplasmic reticulum failure as seen in subjects with newly-detected type 2 diabetes and subjects with impaired glucose tolerance [59]. The generation of insufficient oxidative potential for the formation of disulphide bonds may result in an accumulation of unfolded or misfolded proteins in the lumen of the endoplasmic reticulum, which is referred to as endoplasmic reticulum stress. Endoplasmic reticulum stress has also been found in the tubulointerstitial and glomerular compartments of renal biopsies obtained from patients with diabetic nephropathy [60, 61].

In the kidney, glomerular podocytes are insulin-sensitive cells. Lennon et al. demonstrated that human podocytes cultured in vitro with palmitate lost their insulin-stimulated glucose uptake [62]. The authors suggest that insulin-sensitizing agents such as PPARγ agonists may have direct beneficial effects on podocytes of individuals with type 2 diabetes. Currently, we understand that the ultimate effect of insulin on the transmembrane glucose transport depends on both its plasma concentration and the plasma FFA concentration that determines the degree of membrane flexibility [16, 63–66]. That said, to give more concrete content to the wide-ranging discussion on the type 2 diabetes pathophysiology, we propose to replace the word-combination ‘insulin resistance’ by ‘reduced membrane flexibility’. Finally, in patients with impaired glucose tolerance, the ability of the liver to extract FFA from the circulation is impaired [67]. In this regard, it is of interest that studies have also identified increases in intramyocellular lipid in individuals with an increased relative risk of type 2 diabetes [68]. According to the working hypothesis, tissue hypoxia disturbs mitochondrial β-oxidation of intramyocellular and intrahepatic FFAs.

### Summary

Glycerophospholipids, the major class of naturally occurring membrane phospholipids, have a saturated fatty acid at position 1 and an unsaturated fatty acid at position 2 of the glycerol. In contrast to saturated fatty acids, unsaturated fatty acids have, due to the presence of one or more carbon-carbon double bonds, a non-linear chain. As a result, saturated fatty acids can self-assemble closely together, whereas unsaturated fatty acids prevent such close packing and produce more flexible aggregates. This phenomenon has a great impact on the flexibility of phospholipid membranes. The unsaturation index (UI) is a useful parameter for describing the flexibility of a biological membrane and is calculated by multiplying the mean number of *cis* double bonds per fatty-acid residue by 100. An increased UI implies more membrane flexibility, and a decreased UI implies more membrane stiffness. The released non-esterified fatty-acid pool from human white cells had approximately 110- and 9-fold decreased percentages of docosahexaenoic acid (C22:6) and arachidonic acid (C20:4), respectively, compared to the human serum FFA pool. Thus, an increased release of FFAs from adipose tissue into the circulation, a characteristic of both the prediabetic and the diabetic phase, elevates the plasma concentration of *saturated* fatty acids which results in a shift from unsaturated to saturated fatty-acyl chains in phospholipids of both the erythrocyte membrane and the vascular endothelium. This will cause an increase of diabetes-specific microvascular complications as demonstrated by two recent publications on *Drosophila melanogaster*’s phototransduction, and the synaptic transmission in the visual system of *Drosophila melanogaster* fly’s, and a study on human erythrocyte phospholipid and PUFA composition in diabetic retinopathy. These studies provide new insight about the relationship between impaired membrane flexibility and vascular complications of type 2 diabetes.

## Conclusions

Building on the presented evidence, we suggest that chronically elevated plasma FFAs must be avoided to prevent or postpone reduction of membrane flexibility, which may be the major cause of diabetes-specific complications. We are not concerned with the details of our hypothesis, but we hope that the bigger picture is correct. More research is necessary to better understand the idea of early systemic cell membrane dysfunction.

## Abbreviations

FFA: free fatty acid
PUFA: poly-*cis*-unsaturated fatty acid
UI: unsaturation index
